# Causes of death in preterm neonates (<33 weeks) born in tertiary care hospitals in India: analysis of three large prospective multicentric cohorts

**DOI:** 10.1038/s41372-019-0471-1

**Published:** 2019-09-04

**Authors:** Kajal Jain, M. Jeeva Sankar, Sushma Nangia, Vishnu Bhat Ballambattu, Venkataseshan Sundaram, Siddharth Ramji, Nishad Plakkal, Praveen Kumar, Ashish Jain, Sindhu Sivanandan, Sreenivas Vishnubhatla, Harish Chellani, Ashok Deorari, Vinod K. Paul, Ramesh Agarwal

**Affiliations:** 1 All India Institute of Medical Sciences (AIIMS), New Delhi, India; 2 Lady Hardinge Medical College (LHMC), New Delhi, India; 3 Jawaharlal Institute of Postgraduate Medical Education and Research (JIPMER), Puducherry, India; 4 Postgraduate Institute of Medical Education and Research (PGIMER), Chandigarh, India; 5 Maulana Azad Medical College (MAMC), New Delhi, India; 6 Vardhman Mahavir Medical College and Safdarjung Hospital, New Delhi, India; 7 National Institution for Transforming India (NITI Aayog), Government of India, New Delhi, India

## Abstract

**Objective:**

To estimate the direct causes of mortality among preterm neonates <33 weeks’ gestation by examining three large multicentric, hospital-based datasets in India.

**Method:**

Three prospective hospital-based datasets: National Neonatal Perinatal Database (NNPD) of India, Delhi Neonatal Infection Study (DeNIS) cohort, and Goat Lung Surfactant Extract (GLSE)-Plus cohort were analyzed to study the causes of death among preterm neonates of less than 33 weeks’ gestation admitted to the participating tertiary care hospitals in India.

**Results:**

A total of 8024 preterm neonates were admitted in the three cohorts with 2691 deaths. Prematurity-related complications and sepsis contributed to 53.5% and 19.8% of deaths in the NNPD cohort, 51.0% and 25.0% in the DeNIS cohort, and 39.7% and 40.9% in GLSE-Plus cohort, respectively.

**Conclusions:**

Nearly a quarter (20-40%) of preterm neonates less than 33 weeks’ gestation admitted to Indian NICUs died of sepsis. The study results have implications for health policies targeted to reduce the neonatal mortality rate in India.

## Introduction

Globally, 2.5 million neonates die each year. A vast majority of these deaths occur in low- and middle-income countries (LMICs) [1]. According to recent global estimate of causes of neonatal deaths, prematurity-related complications (35%), intrapartum-related events (birth asphyxia; 23%), and sepsis (27%) accounted for most neonatal deaths [2], 80% of which are preventable with simple interventions [3]. Understanding the cause and timing of neonatal deaths is important to inform public health policies targeted to reduce the neonatal mortality rate (NMR).

The causes of neonatal death vary among countries depending on NMR. In high-income countries (HICs), with lower NMR and high-quality vital registration data, preterm birth and congenital malformations are the most common causes of early (0–6 days) as well as late (7–28 days) neonatal deaths. LMICs with high NMR have incomplete and poor quality vital registration data. These countries often depend on verbal autopsy (VA)-based multicause models to derive causes of neonatal death. According to global [2, 4] and national statistics [5] for causes of death based on VA models, India’s biggest goal to reduce neonatal mortality is reduction in prematurity-related deaths. However, cause of death analysis using VA tools and indirect model based assumptions are fraught with a wide range of uncertainty [6].

In VA models, prematurity-related deaths are defined as deaths resulting from complications of preterm birth, such as surfactant deficiency, intraventricular hemorrhage, necrotizing enterocolitis (NEC), etc. It also includes deaths occurring in neonates <34 weeks of gestation, a level of prematurity below which most preterm birth complications occur, as well as deaths occurring in neonates with birth weight <2000 g for whom gestational age is unknown [4]. This definition might result in the inclusion of term and late preterm neonates who are small-for-gestational age (SGA) as well as preterm neonates with other direct causes of death under the umbrella of prematurity-related deaths. The result is an erroneous inflation of prematurity-related deaths and an underestimation of deaths due to other causes, especially sepsis-related deaths, with important public health implications for resource-limited settings.

South Asian and sub-Saharan African countries contribute to more than 60% of the world’s prematurity burden [6]. The Million Death Study (MDS) [5] recently reported that in India, neonatal deaths from prematurity shows an increasing trend over the last 16 years (2000–2015), whereas deaths due to sepsis and birth asphyxia are decreasing. We speculate that in LMICs, a significant proportion of preterm neonates die of sepsis, contrary to VA-based global estimates that the majority of deaths among preterm neonates is due to prematurity-related complications.

In this paper, we specifically sought to estimate the direct causes of mortality among preterm neonates <33 weeks’ gestation by examining three large multisite, hospital-based datasets in India. We believe that information on cause-specific mortality in preterm neonates will assist in defining appropriate interventions.

## Methods

### Study design

We analyzed three prospective hospital-based datasets: National Neonatal Perinatal Database (NNPD) of India (2002–2003) [7], Delhi Neonatal Infection Study (DeNIS) cohort [8] (2011–2014), and Goat Lung Surfactant Extract (GLSE)-Plus cohort (2016–2017) to study the causes of death among preterm neonates of less than 33 weeks’ gestation admitted to various tertiary care hospitals in India. These datasets were chosen because they involved prospective data collection in a large cohort of inborn neonates, used standardized case definitions, and also provided reliable estimates of underlying cause of death.

#### National Neonatal Perinatal Database (NNPD; 2002–03)

National Neonatal Perinatal database [7] is a large network of 18 tertiary care hospitals across India with level-2 or level-3 neonatal care facilities (names of the sites provided in the Appendix). This network collected data on morbidity and mortality of inborn (born within the center) neonates admitted to participating sites using standardized definitions for data collection and uniform analysis protocol (Table 1). The All India Institute of Medical Sciences (AIIMS), New Delhi acted as the nodal center. The NNPD dataset for the years 2002–2003 provided data on 145,623 live births (Table 2). Term and preterm neonates constituted 84.5 and 14.5% of the cohort, respectively. About half (52.9%) of all live births were males. One third (31.3%) were low birth weight (LBW) and 9.6% were SGA infants. In this cohort, 3680 (2.5%) neonates died in hospital. The NMR of the cohort was 25.3 per 1000 live births. The common causes of neonatal death were perinatal asphyxia (28.8%), prematurity-related complications (26.3%), sepsis (18.6%) and congenital malformations (9.2%). For the purpose of this study, we analyzed the single most important cause of death among 1349 (33.8%) in-hospital deaths from a cohort of 3993 preterm neonates (<33 weeks’ gestation).

**Table 1:** Classification of causes of death as per National Neonatal Perinatal Database (NNPD) definitions

Cause of death	NNPD [7] definition(s)
Perinatal asphyxia	“Death of a neonate in the setting of and with features of perinatal hypoxia and/or birth asphyxia followed by manifestations of or hypoxic ischemic injury of brain (hypoxic ischemic encephalopathy) or other organs.”
Complications related to prematurity	Infants dying of prematurity-related complication such as extremely low gestation, respiratory distress syndrome (RDS), patent ductus arteriosus, bronchopulmonary dysplasia (BPD), necrotizing enterocolitis (NEC), or severe grades (grade III or IV) of intraventricular hemorrhage (IVH).
Neonatal sepsis	Death attributable to pneumonia, sepsis, or meningitis (culture positive as well as culture negative).
Congenital malformations	Death due to lethal congenital malformation.
Others	Cause not classified by above or not established.

**Table 2:** Characteristics of the study datasets and study population included for cause of death analyses

	The National Neonatal Perinatal Database	Delhi Neonatal Infection Study (DeNIS) cohort	Goat Lung Surfactant Extract (GLSE)-Plus cohort*
Consortium composition	18 major tertiary care hospitals across India	Three inborn level-3 NICUs attached to tertiary care teaching hospitals	Five inborn level-3 NICUs attached to tertiary care teaching hospitals
Year of data collection	2002–2003	2011–2014	2016–2017
Place of birth	Inborn neonates	Inborn neonates	Inborn neonates
Description of overall cohort	Live births: 145,623 Neonatal deaths: 3680	Live births: 88,636 Neonates admitted to the NICU for any cause: 14,779 (16.7%)	All neonates admitted to NICU between 26–32 weeks gestation: 2209
Preterm cohort	Preterm neonates <33 weeks’ and <1800 g Live births: 3993 In-hospital deaths: 1349 (33.8%)	Preterm neonates <33 weeks’ and <1800 g Admitted to NICU: 1822 In-hospital deaths: 828 (45.4%)	Preterm neonates 26–32 weeks’ gestation Admitted to NICU: 2209 In-hospital deaths: 514 (23.2%)

*We did not have data on birth weight for GLSE-Plus cohort. Practically, all the babies below 33 weeks are ≤ 1800 g. The proportion of babies with < 26 weeks gestation is also miniscule.

#### DeNIS cohort

Delhi Neonatal Infection Study [8] was a prospective cohort study that examined the incidence, microbiological profile, and antimicrobial resistance (AMR) patterns among cases of neonatal sepsis in three inborn level-3 neonatal units in New Delhi, India, between July 2011 and February 2014 (names of the sites provided in the Appendix). The study was approved by the institutional ethics committees (IEC) of all participating centers. These NICUs had a policy of admitting all neonates <34 weeks or below 1800 g birth weight as well as any sick neonate who required NICU care. Out of 88,636 live births, 14,779 (16.7%) neonates required NICU admission and 13,530 were enrolled in the study (Table 2). The mean (SD) birth weight and gestation of enrolled neonates were 2211 (741) g and 36.0 (3.4) weeks, respectively; approximately two-thirds of neonates were LBW and nearly half were preterm. In the DeNIS cohort, a total of 1822 neonates (13.5%) were born at a gestation age of <33 weeks and weighed <1800 g at birth. Among them 828 (45.4%) died before discharge and were included for the analysis of causes of death.

#### GLSE-Plus cohort

The GLSE study was a randomized controlled trial that compared the safety and efficacy of indigenously manufactured GLSE (Cadisurf^®^, Cadila Pharmaceuticals, India) with bovine surfactant (Survanta^®^ AbbVie, USA) in preterm neonates between 26–32 weeks’ gestation [9]. Five tertiary care NICUs in academic institutions of the country, that catered exclusively to inborn neonates participated in the study from June 2016 to January 2018 (names of the sites provided in the Appendix). The study was approved by the IECs of all participating centers. The five participating study centers pooled the data on the mortality rate and causes of death among all neonates between 26–32 weeks’ gestation admitted in the respective hospitals during the study duration and this constituted GLSE-Plus dataset (Table 2). Among 2209 preterm neonates born at 26–32 weeks’ gestation in the above centers (inclusive of all neonates who were subsequently enrolled in GLSE study), 514 (23.2%) died in hospital and contributed to the analysis of causes of death.

### Assignment of causes of death

For this analysis, we included preterm neonates <33 weeks’ gestation and <1800 g in NNPD and DeNIS datasets and those between 26–32 weeks’ gestation from GLSE-Plus dataset, who died during their birth hospitalization. No exclusion criteria was exercised. Cause of death was medically certified by treating physician (NNPD) or study investigator (DeNIS and GLSE-Plus) using the international medical certificate of cause of death in the format recommended by World Health Organization [10]. The death certificate consists of two parts: Part-1a, states the disease condition directly leading to the cause of death, Part-1b, the antecedent or underlying cause of death (the disease or condition initiating the chain of events leading to death) and Part-2, other significant conditions contributing to death but not related to the disease or condition causing death. The disease or condition filled in Part-1b of the death certificate was assigned as the single most important cause of death.

All datasets classified the underlying cause of death into one of these categories: prematurity-related complications, birth asphyxia or trauma, congenital disorders, sepsis (including pneumonia and meningitis), and used uniform definitions for mortality as put forth by the NNPD collaboration (Table 1). Death was considered to be due to prematurity or its complications if the underlying cause of death was extremely preterm gestation (<28 weeks), respiratory distress syndrome (RDS), bronchopulmonary dysplasia, NEC, patent ductus arteriosus, or severe grades (grade III or IV) of periventricular-intraventricular hemorrhage. Where cause of death was attributed to other conditions like sepsis, asphyxia, or congenital malformations in a preterm neonate, these conditions and not prematurity were considered as the underlying cause. If death could be attributed to two or more problems, the most significant problem as determined by the treating physician was considered.

### Statistical analysis

Cause-specific mortality proportion was calculated using number of deaths until discharge attributed to a specific cause. We calculated pooled estimates and their 95% confidence intervals of each cause of death by taking weighted average of the proportions in the individual cohorts.

## Results

A total of 8024 preterm neonates <33 weeks’ gestation were identified from all the three datasets corresponding to different time periods. Of these 2691 neonates who died in hospital contributed to the analysis of cause of death data (Table 3).

**Table 3:** Single most important cause of death among hospitalized preterm neonates in NNPD, DeNIS, and GLSE-Plus cohorts

Causes of death	NNPD cohort (*N* = 1349)	DeNIS cohort (*N* = 828)	GLSE-Plus cohort (*N* = 514)	Pooled estimates of the proportion of deaths in the three cohorts
Complications related to prematurity	722 (53.5)	423 (51.0)	204 (39.7)	50.1% (48.3–52.0)
Neonatal sepsis	267 (19.8)	207 (25.0)	210 (40.9)	25.4% (23.8–27.0)
Perinatal asphyxia (intrapartum-related events)	166 (12.3)	77 (9.0)	64 (12.5)	11.5% (10.2–12.6)
Congenital malformations	57 (4.2)	23 (3.0)	23 (4.5)	3.8% (3.1–4.6)
Others	137 (10.2)	98 (12.0)	13 (2.5)	9.2% (8.1–10.3)

Values expressed as *Number of deaths* (%)

### NNPD cohort

**Fig. 1:**
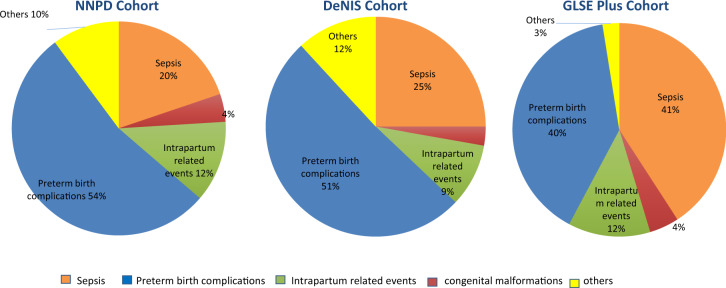
Single most important underlying cause of death in preterm neonates in NNPD (2001–02) cohort, DeNIS (2011–2014) and GLSE-Plus (2016–2017) cohort

Deaths due to preterm birth complications were 53.5% (722/1349) of all deaths, followed by sepsis, accounting for 19.8% (267/1349) (Table 3; Fig. 1). Perinatal asphyxia and malformations accounted for 12.3 and 4.2% respectively.

### DeNIS cohort

Preterm birth complications were the cause of about half (423/828; 51%) of all deaths. Sepsis was the second most common cause, accounting for a quarter of the deaths (207/828; 25%). Perinatal asphyxia and malformations accounted for 9 and 3% of deaths respectively in these neonates (Table 3; Fig. 1).

### GLSE-Plus cohort

Sepsis was the commonest cause of death, implicated in 41% (210/514) of deaths. Prematurity-related complications followed closely, accounting for 39.7% of all deaths (204/514). Perinatal asphyxia and malformations were responsible for 12.5 and 4.5% of deaths respectively in these neonates (Table 3, Fig. 1).

## Discussion

In this study, analyses of three prospective large datasets showed that among preterm neonates of less than 33 weeks’ gestation, sepsis is an important direct cause of death, contributing to 20–40% of mortality. While VA-based estimations would have classified all deaths in this population (<33 weeks’ gestation) to be directly related to prematurity-related complications, hospital-based medical certification attributed the same in only 40–50% of cases. This difference in cause-specific mortality proportion (due to prematurity-related deaths) is important, considering that this information is used to guide public health policy and for targeted allocation of resources. The cause-specific mortality proportion attributed to sepsis varied from 19.8% (NNPD cohort) to 41% (GLSE-Plus cohort). This variation may be explained by differences in patient characteristics, hospital characteristics, and differences in time periods of study. Preventing sepsis-related neonatal deaths in LMICs is important, because two-thirds of all sepsis episodes are due to gram-negative bacterial pathogens, which exhibit a high degree of AMR and are associated with higher case fatality rates [8].

According to the recent MDS report, prematurity is the most important cause of neonatal deaths, accounting for 40% of NMR in India [5]. This study captured 52,252 neonatal deaths over a period of 16 years (from 2000 to 2015) in India and assigned cause-specific mortality based on national sample death registration with VA data. The study also noted an increasing trend in neonatal deaths from prematurity over a 16 year period from 2000 to 2015, while sepsis and intrapartum-related deaths declined. The study highlights that India’s biggest challenge is to tackle prematurity-related deaths in order to progress toward the SDG goal of reducing NMR to 12 per 1000 live births by 2030. However, our analyses inform that about a third of preterm infants die not because of prematurity-related complications but as a result of sepsis.

A death certificate provided by a treating physician is considered the standard for cause of death assignment. In regions where vital registration systems are incomplete or when deaths occur in out-of-hospital settings, VA is the only available tool to ascertain the cause of death. Studies from LMIC settings with high neonatal mortality burdens have shown that VA tools provide a reasonable level of diagnostic accuracy for major causes of neonatal deaths, comparable to medical certification [11, 12]. However, there are potential issues with the case definitions used in VA tools. Deaths are attributed to prematurity by field workers in the community if the neonate was estimated to have a gestational age <33 weeks’ (generally based on last menstrual period date method) or if the neonate was LBW (when gestational age at birth is not known) or just based on a subjective description of “baby very small or smaller than usual” [2, 5]. This misclassification though seemingly unavoidable leads to inflation of prematurity-related deaths and underestimates the incidence of other causes. A study done in rural India based on VA tools estimated that there was a significant overlap among the different causes of death. For example, among 179 neonatal deaths attributed to prematurity, almost a half (95/179) had an overlap with sepsis [13].

There are studies from HICs looking at the cause-specific mortality distribution in preterm population [14–18]. Schindler et al. [14] analyzed the common causes of hospital mortality among preterm neonates <32 weeks’ gestation admitted to Australian NICUs. Among 345 deaths in this population, the most common cause was attributed to prematurity-related complications (includes respiratory conditions, chronic lung disease, IVH, and NEC; 70%), followed by sepsis (16%) and perinatal asphyxia (7.2%). In a German very LBW cohort, 17% (37/221) of in-hospital deaths were attributed to sepsis [18]. A study from various centers affiliated to the National Institute of Child Health and Human Development in the United States [19] examined cause-specific in-hospital mortality among extremely LBW (ELBW) neonates from 2000 to 2011. This study noted that immaturity (gestational age <24 weeks), RDS, and infection were the three common causes of death among ELBW neonates. These studies lend support to our assessment that the primary cause of death needs to be examined in preterm population in LMIC settings too.

Determination of cause of death is essential as they provide programmatic information, since preventive strategies differ based on cause. For preterm birth related complications, the focus is on antenatal corticosteroids and provision of quality NICU care including oxygen, continuous positive air pressure and surfactant. Whereas, to combat sepsis the focus is on asepsis [20, 21], chlorhexidine cord care (in areas with high NMR) [22, 23], appropriate use of antibiotics, and topical emollient (natural plant oils) treatment for hospitalized infants [24]. Other interventions like exclusive breastfeeding, kangaroo care, thermoregulation, etc., influence both. Our study emphasizes that strategies to reduce NMR in India should continue to follow an integrated approach of strengthening family-community care and facility-based services [25].

The strengths of the present study are the use of three multicenter prospective cohorts for cause of death analysis. In all the datasets, a standardized definition for classification of deaths was used and death was certified by the treating physician. The three datasets contributed to a large sample, separated by time and place yet yielded almost similar findings. The following limitations of the study are acknowledged. The use of hospital-based data precludes the generalizability of study results to deaths among preterm neonates in the community, who may fail to receive essential neonatal care. However, with increasing coverage of facility births, most deaths are likely to happen in health care facilities in future. Secondly, assigning the single most important cause of death can be difficult when multiple causes play a role or in facilities with limited diagnostic facilities, however, we have the best possible analysis and assignment of causes of deaths in three cohorts. Since the present study involved secondary analyses of existing data, bias or inaccuracies in cause of death assignment cannot be ruled out. Most participating centers in this analyses are established level-3 NICUs. But many preterm neonates in India are managed in level-2 units (Special Neonatal Care Units) with limited resources [26]. The cause-specific mortality proportion may be different in these settings.

In conclusion, our study shows that nearly a quarter (20-40%) of preterm neonates <33 weeks’ gestation admitted to Indian NICUs die of sepsis. This finding assumes great significance in light of dangerous level of AMR in LMICs. Salvaging these preterm neonates would require optimum strategies to prevent and treat sepsis. The study findings emphasize the need to allocate proportionate resources to tackle the problem of sepsis in these neonates.

## APPENDIX

List of participating centers in NNPD, DeNIS, and GLSE-Plus cohorts

**Table:**

**National Neonatal Perinatal Database of India (NNPD)**
1. All India Institute of Medical Sciences, New Delhi
2. Baroda Medical College & SSG Hospital, Vadodara
3. Christian Medical College and Hospital, Ludhiana
4. Christian Medical College and Hospital, Vellore
5. Government Medical College and Hospital, Chandigarh
6. Indira Gandhi Medical College, Nagpur
7. Institute of Obstetrics and Gynecology, Chennai
8. Jawaharlal Institute of Postgraduate Medical Education and Research (JIPMER), Puducherry
9. King Edward Memorial Hospital, Mumbai
10. King Edward Memorial Hospital, Pune
11. King George Medical College, Lucknow
12. Kasturba Medical College, Manipal
13. Lokmanya Tilak Municipal General Hospital, Sion, Mumbai
14. Maulana Azad Medical College, New Delhi
15. MS Ramaiah Medical College Hospital, Bangalore
16. Postgraduate Institute of Medical Education and Research, Chandigarh
17. St. Johns Medical College and Hospital, Bangalore
18. SVICH, Chennai
**Delhi Neonatal Infection Study (DeNIS Study)**
1. Safdarjung Hospital and Vardhman Mahavir Medical College (VMMC), New Delhi
2. Guruteg Bahadur Hospital and Maulana Azad Medical College (MAMC), New Delhi
3. All India Institute of Medical Sciences (AIIMS), New Delhi
**Goat Lung Surfactant Study (GLSE)-Plus cohort**
1. All India Institute of Medical Sciences (AIIMS), New Delhi, India
2. Lady Hardinge Medical College (LHMC), New Delhi, India
3. Jawaharlal Institute of Postgraduate Medical Education and Research (JIPMER), Puducherry.
4. Postgraduate Institute of Medical Education and Research (PGIMER), Chandigarh.
5. Maulana Azad Medical College (MAMC), New Delhi, India

## References

[1] Lawn JE, Blencowe H, Oza S, You D, Lee AC, Waiswa P (2014). Every newborn: progress, priorities, and potential beyond survival. Lancet.

[2] Liu L, Oza S, Hogan D, Perin J, Rudan I, Lawn JE (2015). Global, regional, and national causes of child mortality in 2000-13, with projections to inform post-2015 priorities: an updated systematic analysis. Lancet.

[3] Lassi ZS, Salam RA, Das JK, Bhutta ZA (2014). Essential interventions for maternal, newborn and child health: background and methodology. Reprod Health.

[4] Oza S, Lawn JE, Hogan DR, Mathers C, Cousens SN (2015). Neonatal cause-of-death estimates for the early and late neonatal periods for 194 countries: 2000-2013. Bull World Health Organ.

[5] Million Death Study Collaborators. (2017). Changes in cause-specific neonatal and 1-59-month child mortality in India from 2000 to 2015: a nationally representative survey. Lancet.

[6] Blencowe H, Cousens S, Oestergaard MZ, Chou D, Moller AB, Narwal R (2012). National, regional, and worldwide estimates of preterm birth rates in the year 2010 with time trends since 1990 for selected countries: a systematic analysis and implications. Lancet.

[7] Neonatal morbidity and mortality: report of the National Neonatal-Perinatal Database. National Neonatology Forum (NNPD Network), India; 2005. www.newbornwhocc.org/pdf/nnpd_report_2002-03.PDF.

[8] Investigators of the Delhi Neonatal Infection Study. (2016). Characterisation and antimicrobial resistance of sepsis pathogens in neonates born in tertiary care centres in Delhi, India: a cohort study. Lancet Glob Health.

[9] Jain Kajal, Nangia Sushma, Ballambattu Vishnu Bhat, Sundaram Venkataseshan, Sankar M. Jeeva, Ramji Siddharth, Vishnubhatla Sreenivas, Thukral Anu, Gupta Yogendra Kumar, Plakkal Nishad, Sundaram Mangalabharathi, Jajoo Mamta, Kumar Praveen, Jayaraman Kumutha, Jain Ashish, Saili Arvind, Murugesan Anitha, Chawla Deepak, Murki Srinivas, Nanavati Ruchi, Rao Suman, Vaidya Umesh, Mehta Ashish, Arora Kamal, Mondkar Jayashree, Arya Sugandha, Bahl Monika, Utture Alpana, Manerkar Swati, Bhat Swarna Rekha, Parikh Tushar, Kumar Manish, Bajpai Anurag, Sivanandan Sindhu, Dhawan Pawandeep Kaur, Vishwakarma Gayatri, Bangera Sudhakar, Kumar Sumit, Gopalakrishnan Shridhar, Jindal Atul, Natarajan Chandra Kumar, Saini Anumeet, Karunanidhi Sukanya, Malik Meenakshi, Narang Parul, Kaur Gurkirat, Yadav Chander Prakash, Deorari Ashok, Paul Vinod K., Agarwal Ramesh (2019). Goat lung surfactant for treatment of respiratory distress syndrome among preterm neonates: a multi-site randomized non-inferiority trial. Journal of Perinatology.

[10] World Health Organization. ICD-11: international statistical classification of diseases and related health problem: eleventh revision. Geneva: WHO; 2018. https://icd.who.int/browse11/l-m/en.

[11] Soofi SB, Ariff S, Khan U, Turab A, Khan GN, Habib A (2015). Diagnostic accuracy of WHO verbal autopsy tool for ascertaining causes of neonatal deaths in the urban setting of Pakistan: a hospital-based prospective study. BMC Pediatr.

[12] Aggarwal AK, Kumar P, Pandit S, Kumar R (2013). Accuracy of WHO verbal autopsy tool in determining major causes of neonatal deaths in India. PLoS One.

[13] Baqui AH, Darmstadt GL, Williams EK, Kumar V, Kiran TU, Panwar D (2006). Rates, timing and causes of neonatal deaths in rural India: implications for neonatal health programmes. Bull World Health Organ.

[14] Schindler T, Koller-Smith L, Lui K, Bajuk B, Bolisetty S, New South W (2017). Causes of death in very preterm infants cared for in neonatal intensive care units: a population-based retrospective cohort study. BMC Pediatr.

[15] Simpson CD, Ye XY, Hellmann J, Tomlinson C (2010). Trends in cause-specific mortality at a Canadian outborn NICU. Pediatrics.

[16] Corchia C, Ferrante P, Da Fre M, Di Lallo D, Gagliardi L, Carnielli V (2013). Cause-specific mortality of very preterm infants and antenatal events. J Pediatr.

[17] Michel MC, Colaizy TT, Klein JM, Segar JL, Bell EF (2018). Causes and circumstances of death in a neonatal unit over 20 years. Pediatr Res.

[18] Stichtenoth G, Demmert M, Bohnhorst B, Stein A, Ehlers S, Heitmann F (2012). Major contributors to hospital mortality in very-low-birth-weight infants: data of the birth year 2010 cohort of the German Neonatal Network. KlinPadiatr.

[19] Patel RM, Kandefer S, Walsh MC, Bell EF, Carlo WA, Laptook AR (2015). Causes and timing of death in extremely premature infants from 2000 through 2011. N Engl J Med.

[20] Agarwal R, Agarwal K, Acharya U, Christina P, Sreenivas V, Seetaraman S (2007). Impact of simple interventions on neonatal mortality in a low-resource teaching hospital in India. J Perinatol.

[21] Balla KC, Rao SP, Arul C, Shashidhar A, Prashantha YN, Nagaraj S (2018). Decreasing central line-associated bloodstream infections through quality improvement initiative. Indian Pediatr.

[22] Sankar MJ, Chandrasekaran A, Ravindranath A, Agarwal R, Paul VK (2016). Umbilical cord cleansing with chlorhexidine in neonates: a systematic review. J Perinatol.

[23] Lassi ZS, Bhutta ZA. Community-based intervention packages for reducing maternal and neonatal morbidity and mortality and improving neonatal outcomes. Cochrane Database Syst Rev. 2015;CD007754. 10.1002/14651858.CD007754.pub3 PMC8498021 25803792

[24] Salam RA, Das JK, Darmstadt GL, Bhutta ZA (2013). Emollient therapy for preterm newborn infants–evidence from the developing world. BMC Public Health.

[25] Ndelema B, Van den Bergh R, Manzi M, van den Boogaard W, Kosgei RJ, Zuniga I (2016). Low-tech, high impact: care for premature neonates in a district hospital in Burundi. a way forward to decrease neonatal mortality. BMC Res Notes.

[26] Neogi SB, Khanna R, Chauhan M, Sharma J, Gupta G, Srivastava R (2016). Inpatient care of small and sick newborns in healthcare facilities. J Perinatol.

